# A Neutrosophic Forecasting Model for Time Series Based on First-Order State and Information Entropy of High-Order Fluctuation

**DOI:** 10.3390/e21050455

**Published:** 2019-05-01

**Authors:** Hongjun Guan, Zongli Dai, Shuang Guan, Aiwu Zhao

**Affiliations:** 1 School of Management Science and Engineering, Shandong University of Finance and Economics, Jinan 250014, China; 2 Courant Institute of Mathematical Sciences, New York University, New York, NY 10012, USA; 3 School of Management, Jiangsu University, Zhenjiang 212013, China

**Keywords:** information entropy, aggregation operator, forecasting, neutrosophic set

## Abstract

In time series forecasting, information presentation directly affects prediction efficiency. Most existing time series forecasting models follow logical rules according to the relationships between neighboring states, without considering the inconsistency of fluctuations for a related period. In this paper, we propose a new perspective to study the problem of prediction, in which inconsistency is quantified and regarded as a key characteristic of prediction rules. First, a time series is converted to a fluctuation time series by comparing each of the current data with corresponding previous data. Then, the upward trend of each of fluctuation data is mapped to the truth-membership of a neutrosophic set, while a falsity-membership is used for the downward trend. Information entropy of high-order fluctuation time series is introduced to describe the inconsistency of historical fluctuations and is mapped to the indeterminacy-membership of the neutrosophic set. Finally, an existing similarity measurement method for the neutrosophic set is introduced to find similar states during the forecasting stage. Then, a weighted arithmetic averaging (WAA) aggregation operator is introduced to obtain the forecasting result according to the corresponding similarity. Compared to existing forecasting models, the neutrosophic forecasting model based on information entropy (NFM-IE) can represent both fluctuation trend and fluctuation consistency information. In order to test its performance, we used the proposed model to forecast some realistic time series, such as the Taiwan Stock Exchange Capitalization Weighted Stock Index (TAIEX), the Shanghai Stock Exchange Composite Index (SHSECI), and the Hang Seng Index (HSI). The experimental results show that the proposed model can stably predict for different datasets. Simultaneously, comparing the prediction error to other approaches proves that the model has outstanding prediction accuracy and universality.

## 1. Introduction

Financial markets are a complex system where fluctuation is the result of combined variables. These variables cause frequent market fluctuations with trends exhibiting degrees of ambiguity, inconsistency, and uncertainty. This pattern implies the importance of time series representations, and thus, an urgent demand arises for analyzing time series data in more detail. To some extent, an effective time series representation can be understood from two aspects: traditional time series prediction approaches [1,2,3,4]; and the fuzzy time series prediction approaches [5,6]. The former emphasizes the use of a crisp set to represent the time series, while the latter uses the fuzzy set.

Generally speaking, data are not only the source for prediction processes or prediction system inputs. The original data, however, are full of noise, incompleteness, and inconsistency, which limit the function of traditional prediction methods. Therefore, Song and Chissom [7,8,9] developed a fuzzy time series model to predict real-time scenarios like college admissions. The fuzzification method effectively eliminates part of the noise inside the data, and the prediction performance of the time series is strengthened. Subsequently, with advancing research, the non-determinacy of information has become the main contradiction affecting prediction accuracy. Some studies proposed novel information representation approaches, such as the type 2 fuzzy time series [5], rough set fuzzy time series [10], and intuitionistic fuzzy time series [11].

Although the above work has achieved considerable results for specific problems, certain shortcomings remain that pose a barrier to the accuracy and applicability of predictions. More specifically, complex scenarios and variables in actual situations make it unrealistic to define and classify explicitly the membership and non-membership of elements.

The neutrosophic sets (NSs) method, proposed by Smarandache [12] for the first time, is suitable for the expression of incomplete, indeterminate, and inconsistent information. A neutrosophic set consists of true-, indeterminacy-, and false-memberships. From the perspective of information representation, scholars have proposed two specific concepts based on the neutrosophic set: single-valued NSs [13] and interval-valued NSs [14]. These concepts are intended to seek a more detailed information representation, thereby enabling NSs to quantify uncertain information more accurately. To deal with the above problem, entropy is an important representation of the degree of the complexity and inconsistency. In a nutshell, entropy is more focused on the representation and measure of inconsistency, while NSs tends to describe uncertainty. Zadeh [15] first proposed the entropy of fuzzy events, which measures the uncertainty of fuzzy events by probability. Subsequently, De Luca and Termin [16] proposed the concept of entropy for fuzzy sets (FSs) based on Shannon’s information entropy theory and further proposed a method of fuzzy entropy measurement. Since information entropy is an effective measurement in the degree of systematic order, it has been gaining popularity for different applications, such as climate variability [17], uncertainty analysis [18,19], financial analysis [20], image encryption [21], and detection [22]. Specifically, He et al. [23] proposed a collapse hazard forecasting method and applied the information entropy measurement to reduce the influence of collapse activity indices. Bariviera [24] proposed a prediction method based on the maximum entropy principle to predict the market and further monitor market anomalies. In Liang’s research [25], information entropy was introduced to analyze trends for capacity assessment of sustainable hydropower development. Zhang et al. [26] proposed a signal recognition theory and algorithm based on information entropy and integrated learning, which applied various types of information entropy including energy entropy and Renyi entropy.

In order to describe the indeterminacy of fluctuations and further measure the inconsistency and uncertainty of dynamic fluctuation trends, we propose a neutrosophic forecasting model based on NSs and information entropy of high-order fuzzy fluctuation time series (NFM-IE). The biggest difference compared to the original models is that the NFM-IE represents both fluctuation trend information and fluctuation consistency information. First of all, a time series is converted to a fluctuation time series by comparing each of the current data and corresponding previous data in the time series. Then, the upward trend of each of the fluctuation data is mapped to the truth-membership of a neutrosophic set and falsity-membership for the downward trend. Information entropy of high-order fluctuation time series is introduced to describe the inconsistency of historical fluctuations and is mapped to the indeterminacy-membership of the neutrosophic set. Finally, an existing similarity measurement method for the neutrosophic set is introduced to find similar states during the forecasting stage, and the weighted arithmetic averaging (WAA) aggregation operator is employed to obtain the forecasting result according to the corresponding similarity. The largest contributions of the proposed model are listed as follows: (1) Introducing information entropy to quantify the inconsistency of fluctuations in related periods and mapping it to the indeterminacy-membership of neutrosophic sets allow NFM-IE to extend traditional forecasting models to a certain level. (2) Employing a similarity measurement method and aggregation operator allows NFM-IE to integrate more possible rules. In order to test its performance, we used the proposed model to forecast some realistic time series, such as the Taiwan Stock Exchange Capitalization Weighted Stock Index (TAIEX), the Shanghai Stock Exchange Composite Index (SHSECI), the Hang Seng Index (HSI), etc. The experimental results show that the model has a stable prediction ability for different datasets. Simultaneously, comparing the prediction error with that from other approaches proves that the model has outstanding prediction accuracy and universality.

The rest of this paper is organized as follows: Section 2 introduces the basic concepts of wave time series and information entropy. Then, the concepts proposed in this paper, such as neutrosophic fluctuation time series (NFTS) and the neutrosophic fluctuation logical relationship, are defined. Section 3 presents the specific modules of the model presented in this paper. Section 4 details the prediction steps and validates the model using TAIEX as the dataset. Section 5 further analyzes the prediction accuracy and universality of the model based on SHSECI and HSI. Finally, the conclusions and prospects are presented in Section 6.

## 2. Preliminaries

### 2.1. Fluctuation Time Series

**Definition** **1.** *Let {V_t_|t = 1, 2, …, T} be a stock time series, where T is the number of observations. Then, {U_t_|t = 2, 3, …, T} is called a fluctuation time series, where U_t_ = V_t_ − V_t−1_ (t = 2, 3, …, T).*

### 2.2. Information Entropy of the m^th^-Order Fluctuation in a Time Series

Information entropy (IE) [27] was proposed as a measurement of event uncertainty. The amount of information can be expressed as a function of event occurrence probability. The general formula for information entropy is:

(1) $$E=-\overset{N}{\underset{t=1}{\sum }}p\left(x_{t}\right)log_{2}\left(p\left(x_{t}\right)\right)$$

where *p(·)* is the probability function of a set of *N* events. In addition, the information entropy must satisfy the following conditions: $\overset{N}{\underset{t=1}{\sum }}p\left(x_{t}\right)=1,\;0<p\left(x_{t}\right)<1$. The information entropy is always positive.

According to the fuzzy set definition by Zadeh [28], each number in a time series can be fuzzified by its membership function of a fuzzy set $L=\left\{L_{1},L_{2},\ldots ,L_{g}\right\}$, which can be regarded as an event in a time series. For example, when *g* = 5, it might represent a set of linguistic event variants as: *L* = {*L*_1_, *L*_2_, *L*_3_, *L*_4_, *L*_5_} *=* {*very low*, *low*, *equal*, *high*, *very high*}, etc.

**Definition** **2.** *Let F(t − 1), F(t − 2), …, F(t − m) be fuzzy sets of the m^th^-order fluctuation time series {U_t_|t = m + 1, m + 2, …, T}. Let p_Ut_(L_1_), p_Ut_ (L_2_), p_Ut_ (L_3_), p_Ut_ (L_4_), and p_Ut_(L_5_) be the probabilities of the occurrence of the linguistic variants L_1_, L_2_, L_3_, L_4_, and L_5_ for F(t − 1), F(t − 2), …, F(t − m). The information entropy of the m^th^-order fluctuation is defined as:*

(2) $$E\left(U_{t}\right)=-\overset{g}{\underset{n=1}{\sum }}p_{Ut}\left(L_{n}\right)\log_{2}\left(p_{Ut}\left(L_{n}\right)\right)$$

*where g = 5, $E\left(U_{t}\right)$ is the information entropy of the m^th^-order fluctuation at point t in the fluctuation time series {U_t_|t = m + 1, m + 2, …, T}.*

### 2.3. Neutrosophic Fluctuation Time Series

**Definition** **3.** *(Smarandache [12]) Let W be a space of points (objects), with a generic element in W denoted by w. A neutrosophic set A in W is characterized by a truth-membership function T_A_(w), am indeterminacy-membership function I_A_(w), and a falsity-membership function F_A_(w). The functions T_A_(w), I_A_(w), and F_A_(w) are real standard or nonstandard subsets of ]0^−^,1^+^[, where $0^{-}=0-\varepsilon$, $1^{+}=1+\varepsilon$, ε>0 is an infinitesimal number. There is no restriction on the sum of T_A_(w), I_A_(w), and F_A_(w).*

**Definition** **4.** *Let {U_t_|t = 2, 3, …, T} be a fluctuation time series of a stock time series as defined in Definition 1. A number U_t_ in U is characterized by an upward-trend function T(U_t_), a fluctuation-inconsistency function I(U_t_), and downward-trend function F(U_t_), which can be correspondingly mapped to the truth-membership, indeterminacy-membership, and falsity-membership dimension of a neutrosophic set, respectively. The upward-trend function T(U_t_) and downward-function F(U_t_) are defined according to the number U_t_ shown as follows:*

(3) $$T(U_{t})=\left\{ \begin{array}{ll} 0, & U_{t}\leq m_{1}^{}\\ f_{1}(U_{t},m_{1}^{},m_{2}^{}), & m_{1}^{}\leq U_{t}\leq m_{2}^{}\\ 1, & otherwise \end{array}\right.\;F(U_{t})=\left\{ \begin{array}{ll} 1, & U_{t}\leq o_{1}^{}\\ f_{2}(U_{t},o_{1}^{},o_{2}^{}), & o_{1}^{}\leq U_{t}\leq o_{2}^{}\\ 1, & otherwise \end{array}\right.$$

*where $m_{j}^{}$ and $o_{j}^{}$ (j = 1, 2) are parameters according to the fluctuation time series.*

The fluctuation-inconsistency function *I*(*U_t_*) can be represented by the information entropy $E\left(U_{t}\right)$ as defined in Equation (2).

Thus, a fluctuation time series {*U_t_|t* = 1, 2, 3, …, *T*} can be represented by a neutrosophic fluctuation time series {*X_t_|t = m* + 1, *m* + 2, …, *T*}, where *X_t_* = (*T*(*U_t_*), *I*(*U_t_*), *F*(*U_t_*)) is a neutrosophic set.

### 2.4. Neutrosophic Logical Relationship

**Definition** **5.** *Let {X_t_|t = 1, 2, 3, …, T} be a fluctuation time series. If there exists a relation R(t, t + 1), such that:*

*X_t_*_+1_ = *X_t_* ◦ *R*(*t*, *t* + 1) (4)

*where ◦ is a max–min composition operator, X_t+1_ is said to be derived from X_t_, denoted by the neutrosophic logical relationship (NLR) X_t_ → X_t+1_. X_t_ and X_t+1_ are called the left-hand side (LHS) and the right-hand side (RHS) of the NLR, respectively. X_t+1_ can also represented by D_t_. Therefore, X_t_ → X_t+1_ can also be represented by X_t_ → D_t_.*

The Jaccard index, also known as the Jaccard similarity coefficient, is used to compare similarities and differences between finite sample sets [29]. The larger the Jaccard similarity value, the higher the similarity.

**Definition** **6.** *Let X_t_, X_j_ be two NSs. The Jaccard similarity between X_t_ and X_j_ in vector space can be expressed as follows:*

(5) $$J\left(X_{t},X_{j}\right)=\frac{T_{X_{t}}T_{X_{j}}+I_{X_{t}}I_{X_{j}}+F_{X_{t}}F_{X_{j}}}{{\left(T_{X_{t}}\right)}^{2}+{\left(I_{X_{t}}\right)}^{2}+{\left(F_{X_{t}}\right)}^{2}+{\left(T_{X_{j}}\right)}^{2}+{\left(I_{X_{j}}\right)}^{2}+{\left(F_{X_{j}}\right)}^{2}-\left(T_{X_{t}}T_{X_{j}}+I_{X_{t}}I_{X_{j}}+F_{X_{t}}F_{X_{j}}\right)}$$

### 2.5. Aggregation Operator for NLRs

**Definition** **7.** *Let $X=\left\{X_{1},X_{2},\ldots ,X_{t},\ldots ,X_{n}\right\}$, $D=\left\{D_{1},D_{2},\ldots ,D_{t},\ldots ,D_{n}\right\}$ be the LHSs and RHSs of a group of NLRs, respectively. The Jaccard similarities between X_t_ (t = 1, 2, …, n) and X_j_ are $S_{X_{i,j}}$ (i = 1, 2, …, n), respectively. The corresponding D_j_ can be calculated by an aggregation operator [30] as:*

(6) $$T_{D_{j}}^{}=\frac{\sum_{t=1}^{n}S_{X_{t,j}}\times T_{D_{t}}^{}}{\sum_{t=1}^{n}S_{X_{t,j}}},\;I_{D_{j}}^{}=\frac{\sum_{t=1}^{n}S_{X_{t,j}}\times I_{D_{t}}^{}}{\sum_{t=1}^{n}S_{X_{t,j}}},$$

*According to the definition of NLR, D_j_ can be represented by X_j+1_*.

## 3. Research Methodology

**Figure 1:**
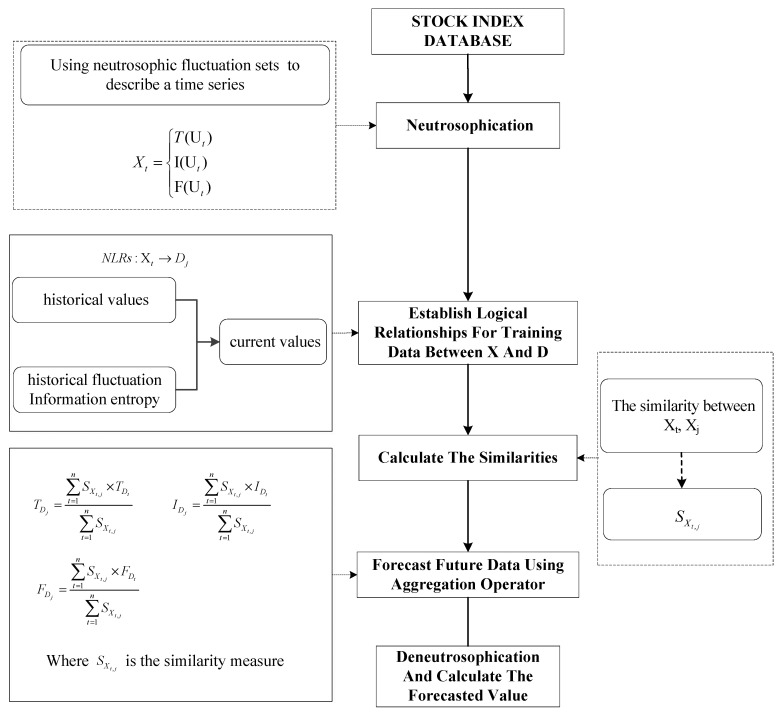
The flowchart of the neutrosophic forecasting model.

In this section, we will introduce a neutrosophic forecasting model for time series based on first-order state and information entropy of high-order fluctuation. The detailed steps are shown as follow steps and in Figure 1.

### 3.1. Step 1: Using Neutrosophic Fluctuation Sets to Describe a Time Series

Let {*V_t_|t* = 1, 2, 3, …, *T*} be a stock index time series and {*U_t_|t* = 2, 3, …, *T*} be its fluctuation time series, where *U_t_ = V_t_ − V_t_*_−1_ *(t* = 2, 3, …, *T)*. Then, we can calculate $len=\frac{\sum_{t=2}^{T}\left|U_{t}\right|}{T-1}$, which is the benchmark for interval division when calculating membership. Let {*X_t_|t = m*, *m* + 1, *m* + 2, …, *T*} be the *m*^th^-order neutrosophic expression of fluctuation time series {*U*_t_*|t* = 2, 3, …, *T*}. The conversion rules for the truth-membership $T_{X_{t}}$ and falsity-membership $F_{X_{t}}$ of *X_t_* are defined as follows:

(7) $$T_{X_{t}}^{}=\left\{ \begin{array}{ll} 0, & U_{t}\leq -0.5len\\ \frac{U_{t}}{3/2\times len}+\frac{1}{3}, & -0.5\times len\leq U_{t}\leq len\\ 1, & U_{t}\geq len \end{array}\right.\;F_{X_{t}}^{}=\left\{ \begin{array}{ll} 1, & U_{t}\leq -len\\ \frac{-U_{t}}{3/2\times len}+\frac{1}{3}, & -len\leq U_{t}\leq 0.5\times len\\ 0, & U_{t}\geq 0.5len \end{array}\right.$$

### 3.2. Step 2: Using Information Entropy to Represent the Complexity of Historical Fluctuations

*{U_t_|t* = 1, 2, 3, …, *T*} can be fuzzified according to a linguistic set *L* = {*l*_1_, *l*_2_, *l*_3_, *l*_4_, *l*_5_}. Specifically, $l_{1}=[U_{\min}\;,\;-1.5\times len)$, $l_{2}=[-1.5\times len\;,\;-0.5\times len)$, $l_{3}=[-0.5\times len\;,\;0.5\times len)$, $l_{4}=[0.5\times len\;,\;1.5\times len)$, and $l_{5}=[1.5\times len\;,\;U_{\max})$. The conversion rule for the indeterminacy-membership $I_{X_{t}}$ is defined as follows:

(8) $$I_{X_{t}}=-\overset{g}{\underset{n=1}{\sum }}p_{X_{t}}\left(L_{n}\right)\log_{2}\left(p_{X_{t}}\left(L_{n}\right)\right)$$

where *g = 5*, $p_{X_{t}}\left(L_{n}\right)$ indicates the probability of occurrence of the label *l_n_* in the past *m* days.

### 3.3. Step 3: Establishing Logical Relationships for Training Data

According to Definition 5, NLRs were established as a training dataset.

### 3.4. Step 4: Calculating the Similarities between Current Data and Training Data

According to Definition 6, similarities between current data and training data were calculated. Let *t* be the current data of the point. $S_{X_{t,j}}$ is the similarity of NFTS between the current point *t* and training data *j*. 

### 3.5. Step 5: Forecasting Neutrosophic Value Using the Aggregation Operator

According to Definition 7, the future neutrosophic fluctuation number $X_{t+1}$ can be generated based on the training dataset and the similarities with $X_{t}$. In order to eliminate very low similarity data, valid NLRs satisfy $S_{X_{t,j}}\geq w^{\prime }$.

### 3.6. Step 6: Deneutrosophication for the Neutrosophic Fluctuation Set and Calculating the Forecasted Value

Calculating the expected value of the forecasted neutrosophic set $X_{t+1}$, the forecasted fluctuation value can be calculated by:

(9) $$V_{t+1}^{\prime }=(T_{X_{t+1}}-F_{X_{t+1}})\times len{+V}_{t}$$

## 4. Empirical Analysis

### 4.1. Prediction Process

#### 4.1.1. Step 1: After Calculating the Fluctuation Value in Stock Time Series, the Fluctuation Values Will Be Converted to Neutrosophic Time Series

This study needs to select the parameters of the model and estimate its performance. Many studies in the field of fuzzy forecasting have used the data from January–October as the training set and the data from November–December as the test dataset. To facilitate comparison with these existing studies, we also selected data from November–December as the test dataset. Considering the characteristics of time series, traditional cross-validation methods (such as *k*-fold cross-validation) have poor adaptability. A subset of data after the training subset needs to be retained for validation of model performance. Therefore, we chose a special nested cross-validation, the outer layer of which was used to estimate the model performance and the inner layer of which was used to select the parameters. Specifically, in this paper, we used TAIEX’s 1999 data as an example. The closing prices from 1 January–31 October were used as the training dataset. Among them, from January–August was a training subset, and from September–October was for validation. Logical relationships were constructed between each dataset and its closest ninth-order historical values. The closing prices from 1 November–31 December were used as forecast data, and performance was evaluated by comparing forecasting and realistic data.

For example, when the fluctuation value is *U*_12_ = 28.7, the sequence of linguistic variables is *l*_4_, *l*_5_, *l*_3_, *l*_3_, *l*_2_, *l*_2_, *l*_2_, *l*_5_, *l*_3_. $p_{U_{12}}(l_{1})$ = 0, $p_{U_{12}}(l_{2})$ = 0.3333, $p_{U_{12}}(l_{3})$ = 0.3333, $p_{U_{12}}(l_{4})$ = 0.1111, $p_{U_{12}}(l_{5})$ = 0.2222. Then, we can calculate the ninth-order fuzzy fluctuation information entropy as follows:

(10) $$E(U_{12})=E(28.7)=-\sum_{i=1}^{5}p_{U_{12}}(l_{i})\log_{2}(p_{U_{12}}(l_{i}))=1.8911$$

(11) $$E(U_{13})=E(-106.5)=-\sum_{i=1}^{5}p_{U_{13}}(l_{i})\log_{2}(p_{U_{13}}(l_{i}))=1.5307$$

(12) $$E(-33.89)=-\sum_{i=1}^{5}p_{U_{14}}(l_{i})\log_{2}(p_{U_{14}}(l_{i}))=1.3923$$

…

The information entropy of fluctuation time proposed in this paper is the intermediate term of NS. In order to maintain the consistency with the other two terms, the above results must be normalized. Normalized information entropy based on the maximum values of information entropy is calculated as follows:

(13) $$E^{\prime }(U_{12})=\frac{1.8911}{3.7000}=0.5111$$

(14) $$E^{\prime }(U_{13})=\frac{1.5307}{3.7000}=0.4137$$

(15) $$E^{\prime }(U_{14})=\frac{1.3923}{3.7000}=0.3763$$

…

In order to convert the numerical data of stock market fluctuation time series into NS, it is necessary to calculate the elements corresponding to the truth-membership term and the falsity-membership term of NS. According to Equation (7), neutrosophic set membership can be calculated. For example, when the fluctuation value is *U*_12_ = 28.7, then truth-membership $T_{X_{12}}$ of *X*_12_ is $\frac{28.7}{3/2\times len}\;+\frac{1}{3}=0.5584$ and falsity-membership $F_{X_{12}}$ of *X*_12_ is $\frac{-28.7}{3/2\times len}\;+\frac{1}{3}=0.1082$. Then, the fluctuation can be represented by the neutrosophic set as follows:

(16) $$X_{12}(28.7)\to (0.5584,0.5111,0.1082)$$

(17) $$X_{13}(-106.5)\to (0.0000,0.4137,1.0000)$$

(18) $$X_{14}(-33.89)\to (0.0675,0.3763,0.5991)$$

…

(19) $$X_{223}(148.18)\to (1.0000,0.3910,0.0000)$$

…

#### 4.1.2. Step 2: According to Definition 5, Establishing Mapping Relationships Based on Historical Values, Historical Trends, and Current Values

This step requires establishing neutrosophic logical relationships based on the feature and target sets, where *X*_12_ is the feature item of *X*_13_.

(20) $$X_{12}(x)\to X_{13}(x)=D_{12}(x)$$

(21) $$X_{13}(x)\to X_{14}(x)=D_{13}(x)$$

…

#### 4.1.3. Step 3: Calculating the Jaccard Similarity

Jaccard similarity is usually used to compare similarities and differences of a limited set of samples. The higher the value, the higher the similarity. We used it to compare the current logical group with the logical groups in the training set in order to identify similar groups. $S_{X_{223,12}}^{\prime }$ indicates the similarity between the 223rd and 12th groups.

(22) $$\begin{array}{ll} S_{X_{223,12}}^{\prime } & =\frac{0.5584\times 1.0000+0.5111\times 0.3910+0.1082\times 0.0000\;}{0.5584^{2}+0.5111^{2}+0.1082^{2}+1.0000^{2}+0.3910^{2}+0.0000^{2}-\left(0.5584\times 1.0000+0.5111\times 0.3910+0.1082\times 0.0000\right)}\\ & =0.7742 \end{array}$$

#### 4.1.4. Step 4: Forecasting the Neutrosophic Fluctuation Point Using the Aggregation Operator

First, we applied the Jaccard similarity measure method to locate similar LHSs of NLRs. We tested different threshold values for the training data. In this example, it was set to 0.89, and we identified 65 groups that met the criteria.

Furthermore, we calculated the forecasting NFTS using the aggregation operator:

D_224_ = (0.5005, 0.5067, 0.3401)

#### 4.1.5. Step 5: Calculating the Forecasted Value

Then, we calculated the predicted fuzzy fluctuation:

(23) $$Y^{\prime }\left(t+1\right)=0.5005-0.3401=0.1604$$

We also calculated the real number of the fluctuation:

(24) $$U^{\prime }\left(t+1\right)=Y^{\prime }\left(t+1\right)\times len=0.1604\times 85=13.63$$

Finally, the predicted value was obtained from the actual value of the previous day and the predicted fluctuation value:

(25) $$V^{\prime }\left(t+1\right)=V\left(t\right)+U^{\prime }\left(t+1\right)=7854.85+13.63=7868.47$$

**Figure 2:**
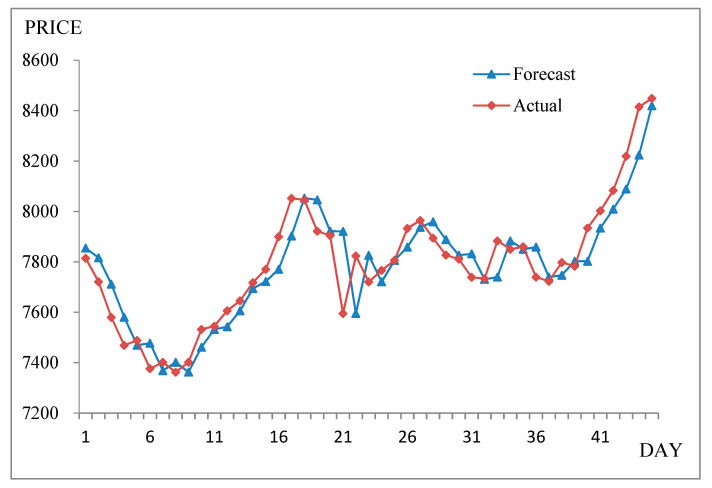
Forecasting results from 1 November 1999–30 December 1999.

For the sample dataset, the complete prediction result of stock fluctuation trends and the actual values are shown in Table 1 and Figure 2.

Table 1 and Figure 2 show that NFM-IE was able to successfully forecast TAIEX data from 1 November 1999–30 December 1999 based on the logical rules derived from training data.

### 4.2. Performance Assessments

During the experimental analysis, some methods were used to measure prediction accuracy in order to quantify model prediction effects. These methods are mainly used in the prediction field, including the mean squared error (MSE), the root mean squared error (RMSE), the mean absolute error (MAE), and the mean absolute percentage error (MAPE).

These expressions are respectively illustrated by Equations (26)–(29):

(26) $$MSE=\frac{\sum_{t=1}^{n}{\left(forecast_{t}-actual_{t}\right)}^{2}}{n}$$

(27) $$RMSE=\sqrt{\frac{\sum_{t=1}^{n}{\left(forecast_{t}-actual_{t}\right)}^{2}}{n}}$$

(28) $$MAE=\frac{\sum_{t=1}^{n}\left|\left(forecast_{t}-actual_{t}\right)\right|}{n}$$

(29) $$MAPE=\frac{\sum_{t=1}^{n}\left|\left(forecast_{t}-actual_{t}\right)\right|/actual_{t}}{n}$$

where *forecast_t_* represents the predicted observations and *actual_t_* represents actual observations.

Theil’s U index [31] is primarily used to measure the deviation between predicted and actual values. It can get a relative value between zero and one, where zero means that the actual value is equal to the predicted value, that is the prediction model is perfect. At the same time, one indicates that the model prediction effect is not satisfactory. Theil’s U index is expressed as follows:

(30) $$U=\frac{\sqrt{\frac{\sum_{t=1}^{n}{\left(forecast_{t}-actual_{t}\right)}^{2}}{n}}}{\sqrt{\frac{\sum_{t=1}^{n}forecast_{t}{}^{2}}{n}}+\sqrt{\frac{\sum_{t=1}^{n}actual_{t}{}^{2}}{n}}}$$

According to Equations (26)–(30), we separately predicted TAIEX data from 1997–2005 and further calculated the error for each year.

From Table 2, the results of different error statistics methods showed that NFM-IE can successfully forecast different time series of TAIEX 1997–2005.

## 5. Results Analysis

### 5.1. Taiwan Stock Exchange Capitalization Weighted Stock Index

In general, TAIEX is a widely-used dataset in stock market forecasting. In order to facilitate comparison with other forecasting models, this paper also uses it as the main dataset to verify the model. Using non-stationary data can lead to spurious regressions, so we first performed a stationarity test based on the unit root test by software Eviews (Eviews10.0 Enterprise Edition, Microsoft, Redmond, WA, USA). It can be concluded that the first-order difference of TAIEX 1997–2005 was stationary data, which indicates that the fluctuation data used in this study were stationary. Further, other datasets in this study were also stationary data.

The model in this paper was based on high order, and thus, different orders may affect the accuracy of the prediction. Hence, the experimental analysis showed that when the order of fuzzy fluctuation information entropy was 9–11, the stability of the model was more ideal. Table 3 shows the experimental errors for different years under different orders.

Not surprisingly, accurate fluctuation trend predictions are very important and needed. Therefore, the performance of different methods must be compared and evaluated, thus verifying the superiority or deficiency of the model. In order to verify the effects of model prediction, this section focuses on comparing this model’s experimental results with those from other models. Comparing the errors across model showed that the current model had certain advantages in prediction accuracy. Table 4 shows the prediction errors for the different methods between 1997 and 2005. The NFM-IE hybrid model achieved better prediction accuracy compared to the traditional regression model, autoregressive model, neural network model, and fuzzy model (Table 4). In addition, NFM-IE exhibited better predictive power in some years compared to other hybrid models based on the fuzzy theory.

### 5.2. Forecasting Shanghai Stock Exchange Composite Index

SHSECI is one of the most typical stock indices in China, with certain representativeness. We selected it as an experimental dataset to verify the model’s applicability. 

**Figure 3:**
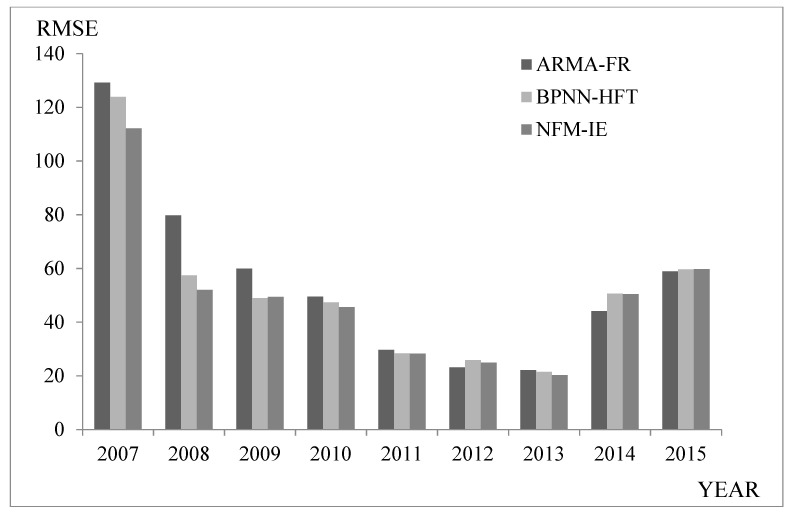
RMSEs of forecast errors for SHSECI from 2007–2015.

Recently, scholars have proposed more comprehensive models based on traditional prediction methods. For example, Guan et al. [39] proposed a two-actor autoregressive moving average model based on the fuzzy logical relationships (ARMA-FR). Guan et al. [40] proposed a model based on back propagation neural network and high-order fuzzy-fluctuation trends (BPNN-HFT). This section compares several typical prediction methods. The results indicated that the model can also effectively predict the stock index. Table 5 and Figure 3 show a comparison of the different prediction methods.

The comparison shows that NFM-IE outperformed other methods in predicting SHSECI from 2007–2015.

Comparing the average value of the SHSECI prediction error showed that NFM-IE had better prediction accuracy and stability compared to the neural network-based BPNN-HFT model and the statistical-based ARMA-FR model.

### 5.3. Forecasting Hong Kong-Hang Seng Index

**Figure 4:**
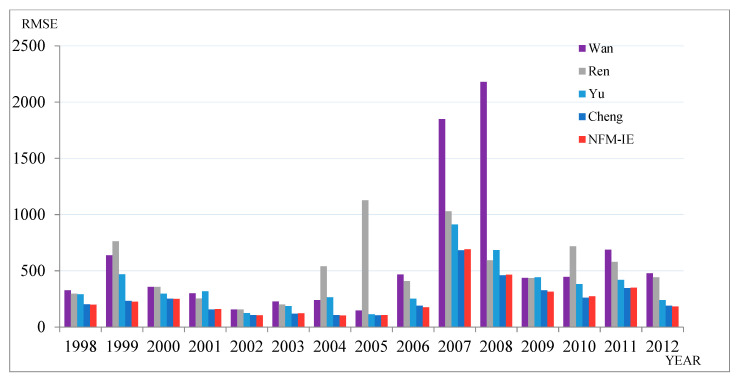
RMSEs of forecast errors for HSI from 1998–2012.

Finally, the Hong Kong-Hang Seng Index (HSI) was selected as the experimental dataset. Comparing several authoritative prediction methods, we can verify the universality of the model in other stock markets. Table 6 and Figure 4 show a comparison of the different prediction methods from 1998–2012.

To further evaluate the validity of the proposed model, we used Friedman’s test to perform a significance test based on the study of Demšar [44]. For reference, Friedman’s test is a parametric statistical test that was proposed by Milton Friedman [45,46]. To further illustrate the significance of the model’s predictions compared to other prediction methods, this section will use Friedman’s test and the post-hoc test for significance analysis. In the Friedman test phase, SPSS was used for statistical testing, and the post-hoc test phase was based on manual calculations.

In the first stage, Friedman’s test requires comparison of the average ranking of different algorithms $R_{j}=\frac{1}{N}\sum_{i}r_{i}^{j}$, where, $r_{i}^{j}$ is the rank of the j-th of k algorithms on the i-th of *N* datasets. The ranking of each method was based on the analysis of HSI forecast results as shown in Table 7.

Through software analysis, we concluded that the method had the highest comprehensive ranking. In addition, according to the Chi-square distribution, there were significant differences between these methods.

(31) $$CD=q_{\alpha }\sqrt{\frac{k(k+1)}{6N}}$$

In the second stage, in order to further compare the different methods, we used the Nemenyi test [47]. According to Equation (31), α = 0.05 and CD = 1.575. Upon further comparison, we found that the method proposed in this study had significant advantages over Yu (2005) [41], Wan (2017) [42], Ren (2016) [43], etc. Although it was not significant compared with Cheng’s method (2018) [10], the NFM-IE had certain advantages from the perspective of error mean and average level.

### 5.4. Discussion

The research was mainly focused on two issues. The first was whether the uncertainty of stock market volatility can be used as a key feature of forecasting in a complex environment. The other was whether the prediction method considering uncertainty and trend was effective. We first used the inconsistency of historical fluctuations as a stock forecasting feature and further characterized and quantified it. Then, we applied the neutrosophic set to be the representation of the information and established a neutrosophic logic relationship based on wave inconsistency. Through experimental analysis, the proposed model achieved robustness and stability with relatively few parameters. In addition, it was also proven that predictions that consider inconsistency are meaningful and effective. The advantages were embodied in the following aspects: First, NFM-IE did not need to establish complex assumptions compared to traditional regression-based prediction models. Second, the NFM-IE prediction process was more interpretable than the neural network. Finally, compared with the fuzzy prediction method, NFM-IE effectively utilized data inconsistency as key information. All in all, the model showed satisfactory performance. However, it also showed certain limitations: First, the model used single stock market data as the system input and failed to consider multiple factors fully. Secondly, using information entropy as a key tool for uncertainty measurement requires further optimization in characterizing data.

## 6. Conclusions

In this paper, we presented the concept of NFTS and proposed a prediction model based on the neutrosophic set and information entropy of high-order fuzzy fluctuation time series. This model had significant performance advantages over existing fuzzy time series models, machine learning prediction models, and traditional economic prediction models. In this paper, we applied three typical test datasets to prove that the model had certain universality and stability. In addition, this paper had a certain degree of scientific contribution in the following aspects: First, the concept of NFTS was proposed. Second, this paper proposed information entropy based on high-order fluctuation time series. Finally, this paper established NLRs based on NFTS and information entropy. This paper discussed the first-order neutrosophic time series to characterize the historical state of uncertainty and high-order information fluctuation entropy to measure the complexity of historical fluctuations. Other types of time series will be tested in the future. Meanwhile, future research should aim to establish detailed high-order neutrosophic time series models indicating the uncertainty of historical trends. In this study, we have considered the Jaccard similarity measure for comparing *X_t* and *X_j*. Further work could considered the Jensen–Shannon distance [20], which accomplishes the triangular inequality. Furthermore, in order to verify the robustness of the forecast in longer forecast scenarios, we will extend the model to 2, 3, or 4 periods ahead.

## Figures and Tables

**Table 1:** Forecasting results from 1 November 1999–30 December 1999.

Date (MM/DD/YYYY)	Actual	Forecast	(Forecast − Actual)^2^	Date (MM/DD/YYYY)	Actual	Forecast	(Forecast − Actual)^2^
11/1/1999	7814.89	7868.47	2871.08	12/1/1999	7766.20	7719.40	2190.11
11/2/1999	7721.59	7821.82	10,046.31	12/2/1999	7806.26	7770.62	1270.07
11/3/1999	7580.09	7722.04	20,149.71	12/3/1999	7933.17	7814.75	14,022.27
11/4/1999	7469.23	7577.92	11,813.96	12/4/1999	7964.49	7944.99	380.16
11/5/1999	7488.26	7466.90	456.14	12/6/1999	7894.46	7968.41	5468.57
11/6/1999	7376.56	7489.54	12,764.37	12/7/1999	7827.05	7895.11	4631.50
11/8/1999	7401.49	7374.68	718.73	12/8/1999	7811.02	7826.02	225.13
11/9/1999	7362.69	7399.02	1320.19	12/9/1999	7738.84	7808.59	4864.78
11/10/1999	7401.81	7371.66	909.13	12/10/1999	7733.77	7738.76	24.94
11/11/1999	7532.22	7391.20	19,887.04	12/13/1999	7883.61	7723.92	25,501.56
11/15/1999	7545.03	7543.08	3.82	12/14/1999	7850.14	7897.06	2201.62
11/16/1999	7606.20	7536.55	4851.14	12/15/1999	7859.89	7854.28	31.42
11/17/1999	7645.78	7613.89	1017.07	12/16/1999	7739.76	7860.82	14,654.64
11/18/1999	7718.06	7643.21	5603.26	12/17/1999	7723.22	7738.34	228.50
11/19/1999	7770.81	7729.37	1716.87	12/18/1999	7797.87	7722.01	5754.66
11/20/1999	7900.34	7780.44	14,376.84	12/20/1999	7782.94	7811.00	787.09
11/22/1999	8052.31	7915.24	18,788.73	12/21/1999	7934.26	7782.84	22,929.50
11/23/1999	8046.19	8068.19	483.82	12/22/1999	8002.76	7946.35	3182.30
11/24/1999	7921.85	8046.12	15,443.79	12/23/1999	8083.49	8016.21	4526.63
11/25/1999	7904.53	7919.37	220.29	12/24/1999	8219.45	8096.51	15,113.68
11/26/1999	7595.44	7906.37	96,679.93	12/27/1999	8415.07	8233.25	33,058.13
11/29/1999	7823.90	7592.64	53,479.11	12/28/1999	8448.84	8429.73	365.06
11/30/1999	7720.87	7836.52	13,376.00	Root Mean Square Error (RMSE)			102.02

**Table 2:** Comparing results of different error statistics methods for Taiwan Stock Exchange Capitalization Weighted Stock Index (TAIEX) data collected from 1997–2005.

Year	1997	1998	1999	2000	2001	2002	2003	2004	2005
RMSE	141.42	114.69	102.02	129.94	114.22	66.84	53.88	55.24	53.1
MSE	19,999.62	13,153.80	10,408.08	16,884.40	13,046.21	4467.59	2903.05	3051.46	2819.61
MAE	113.42	96.31	79.38	96.65	92.48	51.65	41.11	38.65	41.27
MAPE	0.0143	0.0138	0.0102	0.0182	0.019	0.0111	0.007	0.0065	0.0067
Theil’s U	0.0089	0.0082	0.0065	0.0122	0.0119	0.0072	0.0046	0.0047	0.0043

**Table 3:** Comparing average RMSEs based on different order fuzzy fluctuation time series from 1997–2005.

Order	8	9	10	11	12	13	14	15	16
1997	141.41	141.42	141.46	141.9	141.53	141.72	141.68	141.8	141.69
1998	114.67	114.69	114.61	114.76	114.63	114.39	114.46	114.29	114.23
1999	101.86	102.02	101.7	101.66	101.55	101.59	101.7	101.26	101.54
2000	129.07	129.94	129.62	129.34	129.87	129.49	128.64	128.6	128.43
2001	113.97	114.22	114.53	114.86	115.37	115.11	115.39	116.06	116.02
2002	67.29	66.84	66.95	66.85	66.76	67.21	66.98	67.02	67.48
2003	53.84	53.88	53.99	53.68	53.74	53.8	53.55	53.48	53.45
2004	54.7	55.24	55.17	55.08	55.07	55.36	55.47	55.1	55.25
2005	53.09	53.1	53.22	53.09	53.14	53.11	53.13	53.04	52.97
average	92.21	92.37	92.36	92.36	92.41	92.42	92.33	92.29	92.34
total	829.9	831.35	831.25	831.22	831.66	831.78	831	830.65	831.06

**Table 4:** Performance comparison of prediction RMSEs with other models. NFM-IE, neutrosophic forecasting model based on information entropy.

TYPE	Methods	RMSE										
		1997	1998	1999	2000	2001	2002	2003	2004	2005	Average	Total
Regression Model	Univariate conventional regression model (U_R) [32,33]	N/A	N/A	164	420	1070	116	329	146	N/A	374.20	2245
	Bivariate conventional regression model (B_R) [32,33]	N/A	N/A	103	154	120	77	54	85	N/A	98.80	593
Auto-regressive	Autoregressive model for order one (AR_1) [34]	146.22	144.53	116.84	155.12	**112.39**	97.09	91.67	79.94	N/A	117.98	653.05
	Autoregressive model for order two (AR_2) [34]	174.09	135.21	128.15	142.3	129.84	89.8	66.58	60.33	N/A	115.79	617
Neural network	Univariate neural network model (U_NN) [32,33]	N/A	N/A	107	309	259	78	57	60	N/A	145.00	870
	Bivariate neural network mode (B_NN) [32,33]	N/A	N/A	112	274	131	69	**52**	61	N/A	116.40	699
Fuzzy	fuzzy forecasting and fuzzy rule(F-R) [35]	N/A	N/A	123.64	131.1	115.08	73.06	66.36	60.48	N/A	94.95	569.72
	Fuzzy time-series model based on rough set rule (F-RS) [10]	N/A	120.8	110.7	150.6	113.2	66	53.1	58.6	53.5	**90.81**	605.7
	Fuzzy variation groups (F-VG) [36]	140.86	144.13	119.32	129.87	123.12	71.01	65.14	61.94	N/A	106.92	570.4
Fuzzy+	Multi-variable fuzzy and particle swarm optimization (M_F-PSO) [37]	**138.41**	113.88	102.34	131.25	113.62	**65.77**	52.23	56.16	N/A	96.71	521.37
	Univariate fuzzy and particle swarm optimization (U_F-PSO) [38]	143.6	115.34	99.12	**125.7**	115.91	70.43	54.26	57.24	54.68	92.92	577.34
	Autoregressive moving average and fuzzy logical Relationships (ARMA-FR) [39]	141.89	119.85	99.03	128.62	125.64	66.29	53.2	56.11	55.83	94.05	584.72
	Back propagation neural network and high-order fuzzy-fluctuation trends (BPNN-HFT) [40]	142.99	**112.51**	**96.77**	126.85	120.12	66.39	54.87	58.1	54.7	92.59	577.8
	NFM-IE	141.42	114.69	102.02	129.94	114.22	66.84	53.88	**55.24**	**53.1**	92.37	**575.24**

**Table 5:** RMSEs of forecast errors for the Shanghai Stock Exchange Composite Index SHSECI from 2007–2015.

Year	2007	2008	2009	2010	2011	2012	2013	2014	2015	Average
ARMA-FR (2017) [39]	129.22	79.77	59.96	49.48	29.7	**23.14**	22.13	**44.11**	**58.89**	55.15
BPNN-HFT (2018) [40]	123.89	57.44	**48.92**	47.34	28.37	25.84	21.43	50.59	59.69	51.50
NFM-IE	**112.10**	**51.98**	49.37	**45.58**	**28.22**	24.92	**20.21**	50.44	59.77	**49.17**

**Table 6:** RMSEs of forecast errors for the Hong Kong-Hang Seng Index (HSI) from 1998–2012.

Method	1998	1999	2000	2001	2002	2003	2004	2005	2006	2007	2008	2009	2010	2011	2012	Average
Yu (2005) [41]	291.4	469.6	297.05	316.85	123.7	186.16	264.34	112.4	252.44	912.67	684.9	442.64	382.06	419.67	239.11	359.66
Wan (2017) [42]	326.62	637.1	356.7	299.43	155.09	226.38	239.63	147.2	466.24	1847.8	2179	437.24	445.41	688.04	477.34	595.26
Ren (2016) [43]	296.67	761.9	356.81	254.07	155.4	199.58	540.19	1127	407.89	1028.7	593.8	435.18	718.33	578.7	442.44	526.46
Cheng (2018) [10]	201.99	231.91	251.7	156.58	106.26	118.74	105.38	103.96	189.2	682.08	460.12	326.65	260.67	346.33	190.13	248.78
NFM-IE	**195.86**	**223.91**	**246.11**	163.49	**105.65**	122.04	**102.23**	105.37	**173.55**	694.89	469.11	**319.7**	274.73	347.2	**181.98**	248.39

**Table 7:** The rank of the forecasting results of the HSI.

Method	Rank
Yu (2005) [41]	3.40
Wan (2017) [42]	4.40
Ren (2016) [43]	4.20
Cheng (2018) [10]	1.53
NFM-IE	1.47

## References

[1] Han M., Xu M. (2018). Laplacian Echo State Network for Multivariate Time Series Prediction. IEEE Trans. Neural Netw. Learn. Syst..

[2] Mishra N., Soni H.K., Sharma S., Upadhyay A.K. (2018). Development and Analysis of Artificial Neural Network Models for Rainfall Prediction by Using Time-Series Data. Int. J. Intell. Syst. Appl..

[3] Safari N., Chung C.Y., Price G.C.D. (2018). A Novel Multi-Step Short-Term Wind Power Prediction Framework Based on Chaotic Time Series Analysis and Singular Spectrum Analysis. IEEE Trans. Power Syst..

[4] Moskowitz D. (2018). Implementing the template method pattern in genetic programming for improved time series prediction. Genet. Program. Evol. Mach..

[5] Soto J., Melin P., Castillo O. (2018). Ensembles of Type 2 Fuzzy Neural Models and Their Optimization with Bio-Inspired Algorithms for Time Series Prediction.

[6] Soares E., Costa P., Costa B., Leite D. (2018). Ensemble of evolving data clouds and fuzzy models for weather time series prediction. Appl. Soft Comput..

[7] Song Q., Chissom B.S. (1993). Forecasting enrollments with fuzzy time series—Part I. Fuzzy Sets Syst..

[8] Song Q., Chissom B.S. (1993). Fuzzy time series and its models. Fuzzy Sets Syst..

[9] Song Q., Chissom B.S. (1991). Forecasting enrollments with fuzzy time series—Part II. Fuzzy Sets Syst..

[10] Cheng C.H., Yang J.H. (2018). Fuzzy Time-Series Model Based on Rough Set Rule Induction For Forecasting Stock Price. Neurocomputing.

[11] Kumar S., Gangwar S. (2016). Intuitionistic fuzzy time series: An approach for handling non-determinism in time series forecasting. IEEE Trans. Fuzzy Syst..

[12] Smarandache F. (1999). A unifying field in logics: Neutrosophic logic. Mult.-Valued Log..

[13] Wang H., Smarandache F., Zhang Y.Q., Sunderraman R. (2010). Single valued neutrosophic sets. Multispace Multistruct.

[14] Wang H., Smarandache F., Zhang Y.Q., Sunderraman R. (2005). Interval Neutrosophic Sets and Logic: Theory and Applications in Computing.

[15] Zadeh L.A. (1968). Probability measure of fuzzy events. J. Math. Anal. Appl..

[16] DeLuca A.S., Termini S. (1972). A definition of nonprobabilistic entropy in the setting of fuzzy set theory. Inf. Control.

[17] Vu T.M., Mishra A.K., Konapala G. (2018). Information Entropy Suggests Stronger Nonlinear Associations between Hydro-Meteorological Variables and ENSO. Entropy.

[18] Zeng X., Wu J., Wang D., Zhu X., Long Y. (2016). Assessing Bayesian model averaging uncertainty of groundwater modeling based on information entropy method. J. Hydrol..

[19] Arellano-Valle R.B., Contreras-Reyes J.E., Stehlík M. (2017). Generalized skew-normal negentropy and its application to fish condition factor time series. Entropy.

[20] Liu Z., Shang P. (2018). Generalized information entropy analysis of financial time series. Physica A.

[21] Ye G., Pan C., Huang X., Zhao Z., He J. (2018). A Chaotic Image Encryption Algorithm Based on Information Entropy. Int. J. Bifurcation Chaos.

[22] Tang Y., Liu Z., Pan M., Zhang Q., Wan C., Guan F., Wu F., Chen D. (2018). Detection of Magnetic Anomaly Signal Based on Information Entropy of Differential Signal. IEEE Geosci. Remote Sens. Lett..

[23] He H., An L., Liu W., Zhang J. (2017). Prediction Model of Collapse Risk Based on Information Entropy and Distance Discriminant Analysis Method. Math. Prob. Eng..

[24] Bariviera A.F., Martín M.T., Plastino A., Vampa V. (2016). LIBOR troubles: Anomalous movements detection based on maximum entropy. Physica A.

[25] Liang X., Si D., Xu J. (2018). Quantitative Evaluation of the Sustainable Development Capacity of Hydropower in China Based on Information Entropy. Sustainability.

[26] Zhang Z., Li Y., Jin S., Zhang Z., Wang H., Qi L., Zhou R. (2018). Modulation Signal Recognition Based on Information Entropy and Ensemble Learning. Entropy.

[27] Shannon C.E. (1948). A mathematical theory of communication. Bell Labs Tech. J..

[28] Zadeh L.A. (1974). The Concept of a Linguistic Variable and its Application to Approximate Reasoning. Inf. Sci..

[29] Fu J., Ye J. (2017). Simplified neutrosophic exponential similarity measures for the initial evaluation/diagnosis of benign prostatic hyperplasia symptoms. Symmetry.

[30] Ali M., Son L.H., Thanh N.D., Minh N.V. (2017). A neutrosophic recommender system for medical diagnosis based on algebraic neutrosophic measures. Appl. Soft Comput..

[31] Theil H. (1966). Applied Economic Forecasting.

[32] Yu T.H.K., Huarng K.H. (2008). A bivariate fuzzy time series model to forecast the TAIEX. Expert Syst. Appl..

[33] Yu T.H.K., Huarng K.H. (2010). Corrigendum to ‘‘A bivariate fuzzy time series model to forecast the TAIEX”. Expert Syst. Appl..

[34] Sullivan J., Woodall W.H. (1994). A comparison of fuzzy forecasting and Markov modeling. Fuzzy Sets Syst..

[35] Chen S.M., Chang Y.C. (2010). Multi-variable fuzzy forecasting based on fuzzy clustering and fuzzy rule interpolation techniques. Inf. Sci..

[36] Chen S.M., Chen C.D. (2011). TAIEX Forecasting Based on Fuzzy Time Series and Fuzzy Variation Groups. IEEE Trans. Fuzzy Syst..

[37] Chen S.M., Manalu G.M., Pan J.S., Liu H.C. (2013). Fuzzy Forecasting Based on Two-Factors Second-Order Fuzzy-Trend Logical Relationship Groups and Particle Swarm Optimization Techniques. IEEE Trans. Cybern..

[38] Jia J., Zhao A.W., Guan S. (2017). Forecasting Based on High-Order Fuzzy-Fluctuation Trends and Particle Swarm Optimization Machine Learning. Symmetry.

[39] Guan S., Zhao A. (2017). A Two-Factor Autoregressive Moving Average Model Based on Fuzzy Fluctuation Logical Relationships. Symmetry.

[40] Guan H., Dai Z., Zhao A. (2018). A novel stock forecasting model based on High-order-fuzzy-fluctuation Trends and Back Propagation Neural Network. PLoS ONE.

[41] Yu H.K. (2005). A refined fuzzy time-series model for forecasting. Physica A.

[42] Wan Y., Si Y.W. (2017). Adaptive neuro fuzzy inference system for chart pattern matching in financial time series. Appl. Soft Comput..

[43] Ren Y., Suganthan P.N., Srikanth N. (2016). A Novel Empirical Mode Decomposition With Support Vector Regression for Wind Speed Forecasting. IEEE Trans. Neural Netw. Learn. Syst..

[44] Demšar J. (2006). Statistical comparisons of classifiers over multiple datasets. J. Mach. Learn. Res..

[45] Friedman M. (1937). The use of ranks to avoid the assumption of normality implicit in the analysis of variance. J. Am. Stat. Assoc..

[46] Friedman M. (1940). A comparison of alternative tests of significance for theproblem of m rankings. Ann. Math. Stat..

[47] Nemenyi P. (1963). Distribution-free Multiple Comparisons. Ph.D. Thesis.

